# Integration of cellular ubiquitin and membrane traffic systems: focus on deubiquitylases

**DOI:** 10.1111/febs.14007

**Published:** 2017-01-29

**Authors:** Michael J. Clague, Sylvie Urbé

**Affiliations:** ^1^ Cellular and Molecular PhysiologyInstitute of Translational Medicine, University of LiverpoolUK

**Keywords:** autophagy, deubiquitylase, endocytosis, mitophagy, ubiquitin

## Abstract

The cell is comprised of integrated multilevel protein networks or systems. The ubiquitin, protein homeostasis and membrane trafficking systems are highly integrated. Here, we look at the influence of reversible ubiquitylation on membrane trafficking and organelle dynamics. We review the regulation of endocytic sorting, selective autophagy and the secretory pathway by ubiquitin signals, with a particular focus on detailing the contribution of deubiquitylating enzymes.

**Abbreviations**
ALG2: asparagine‐linked glycosylation protein 2 homolog
AMSH: associated molecule with the SH3‐domain of STAM
ATG: autophagy‐related gene/protein
BTB: Broad‐Complex, Tramtrack and Bric a brac domain
Cbl: casitas B‐lineage lymphoma proto‐oncogene
CFTR: cystic fibrosis transmembrane conductance regulator
ciM6PR: cation‐independent mannose 6‐phosphate receptor
CRL: cullin‐RING ubiquitin ligase
DUB: deubiquitylase or deubiquitinase
E1: ubiquitin‐activating enzyme
E2: ubiquitin‐conjugating enzyme
E3: ubiquitin ligase
EGFR: epidermal growth factor receptor
ELDR: endo‐lysosomal damage response
EPS15: epidermal growth factor receptor pathway substrate 15
ERAD: ER‐associated degradation
ER: endoplasmic reticulum
ESCRT: endosomal sorting complex required for transport
Fz: frizzled
GABARAP: GABA(A) receptor‐associated protein
GFP: green fluorescent protein
GPCR: G protein‐linked receptors
HACE1: HECT domain and ankyrin repeat containing e3 ubiquitin protein ligase 1
HECT: Homologous to the E6‐AP carboxyl terminus
HRS (HGS): hepatocyte growth factor‐regulated tyrosine kinase substrate
IDOL: inducible degrader of the LDL‐receptor
ILVs: intralumenal vesicles
LC3: light chain 3
LDLR: low‐density lipoprotein (LDL) receptor
LRP6: LDL receptor‐related protein 6
MAPS: misfolding‐associated protein secretion
MIC‐CAP: microcephaly capillary malformation syndrome
MIT: microtubule interacting and transport domain
MVB: multivesicular body
MYLIP: myosin regulatory light chain‐interacting protein
NEDD4: neural precursor cell expressed, developmentally down‐regulated 4
PDGFR: platelet‐derived growth factor receptor
PD: Parkinson's disease
PEF1: penta‐EF‐hand domain containing 1
PINK1: PTEN‐induced putative kinase 1
PLAA: phospholipase A2‐activating protein
PTEN: phosphatase and tensin homologue
PTP1B: protein tyrosine phosphatase, nonreceptor type 1
pUb: Ubiquitin phosphorylated on Ser65
RING: really interesting new gene
RNF26: ring finger protein 26
ROS: reactive oxygen species
RTK: receptor tyrosine kinase
Smo: smoothened
SNARE: soluble N‐ethylmaleimide attachment protein receptor
SNCA: α‐synuclein
SNX1: sorting nexin 1
SPOPL: speckle Type BTB/POZ protein like
SQSTM1: sequestosome 1
STAM: signal‐transducing adaptor molecule
TCRα: T‐cell receptor‐α
TGN: trans‐Golgi Network
TOLLIP: toll‐interacting protein
TRIM27: tripartite motif containing 27
Ubl: ubiquitin‐like domain
USP: ubiquitin‐specific protease
VCIP135: valosin‐containing protein‐interacting protein 1
VEGFR: vascular endothelial growth factor receptor
VHL: von‐hippel lindau
VPS: vacuolar protein sorting
VSV G: vesicular stomatitis virus glycoprotein
WASH: WASH and scar homolog

## Introduction

The complement of expressed proteins provides the building blocks for an integrated set of systems that combine to orchestrate overall cell behaviour. The ubiquitin system constitutes > 1.5% of cellular protein. It overlaps with, not only the proteostasis system but also with the cell signalling system, by generating a complex signalling code on 1000's of substrate proteins 1, 2, 3. The spectrum of ubiquitylated proteins in a cell is determined by the net activity of ubiquitin conjugation cascades (involving E1s, E2s and E3s), which principally attach ubiquitin molecules to lysine residues, balanced by the opposing action of the deubiquitylating enzymes (DUBs), which hydrolyse this isopeptide bond. The seven internal lysines of ubiquitin allow for the formation of topologically distinct ubiquitin isopeptide chain types. These, together with a minor fraction of linear ubiquitin chains and a large fraction of mono‐ubiquitylation sites may be recognised by multiple types of reader proteins that contain ubiquitin‐binding domains that effectively translate the ubiquitin code 4, 5. Some E2s, DUBs and readers display exquisite selectivity for particular ubiquitin chain linkage types, while others show little discrimination. The length of an ubiquitin chain is also an important parameter that can determine avidity for lower affinity binders. The cell membrane traffic system controls protein turnover through endocytosis and autophagy, but more fundamentally maintains compartmental organisation in the face of large‐scale membrane flux. Here, we will discuss the integration of the ubiquitin and membrane trafficking systems.

Subcellular compartmentalisation results from the differential trafficking patterns of membrane components. From the point of view of ubiquitin‐conjugating and deubiquitylating enzymes, it provides a means to confer specificity by restricting the palette of available substrates. Thus, understanding of the ubiquitin system may require localisation data for all components. To date, systematic subcellular localisation has been reported for the majority of DUBs and for the fraction of RING E3 ligases possessing a *trans*‐membrane domain 6, 7, 8, 9. In this review, we will explore the impact of reversible ubiquitylation on membrane trafficking and organelle dynamics.

## Ubiquitin as a sorting signal

The distribution of membrane proteins is largely governed through a balance of active sorting and retention signals, which governs their inclusion into vesicular or tubular transport intermediates 10. For the most part their flow is constitutive, determined by intrinsic sequence‐mediated signals (e.g. KDEL for ER retention, NPXY for endocytosis of LDL receptor). Cells also reconfigure the subcellular distribution of proteins in response to prevailing conditions. In many cases, ligand occupancy of receptors is coupled to their redistribution, which is accomplished through the generation of sorting signals by post‐translational modification. Although ubiquitylation can serve as a supplementary plasma membrane internalisation signal, for numerous receptors its most critical function resides at the sorting endosome. Here, it promotes receptor incorporation into intralumenal vesicles (ILVs) of the multivesicular body (MVB) which commits cargo for lysosomal degradation 11.

Early work in the 1990s identified ubiquitylation of a number of plasma membrane proteins but its significance for membrane trafficking only became clear from pioneering studies in yeast 12, 13, 14. Shortly thereafter, studies of *Caenorhabditis elegans* vulval development led to the identification of SLI‐1, a homologue of the ubiquitin E3‐ligase c‐Cbl, as a negative regulator of signalling downstream of the LET‐23 receptor tyrosine kinase (RTK) 15. Extension of these results to human cells quickly followed, with the observation that epidermal growth factor receptor (EGFR) recruits c‐Cbl to endosomes leading to EGFR ubiquitylation and receptor down‐regulation through lysosomal sorting 16. Mass spectrometry studies suggest that epidermal growth factor receptor (EGFR) is ubiquitylated at multiple sites, with Lys63‐linked chains highly represented and necessary for sorting 17, 18. A prominent role for Lys63‐linked ubiquitin chains in the endosomal pathway is also evident in yeast 19. c‐Cbl‐mediated ubiquitylation was established as a widely used sorting signal that promotes lysosomal sorting of multiple activated RTKs (e.g. EGFR, c‐MET, PDGFR) 20. There are exceptions, for example, ITCH was identified as the major E3‐ligase dictating degradation of the EGFR family member, ERBB4 21. Other classes of receptors such as chronically activated G protein‐coupled receptors (GPCRs) use ubiquitylation for lysosomal down‐regulation, but the cognate E3s are likely more variable (e.g. NEDD4 ubiquitylates β2‐adrenergic receptor 22). Under conditions of excess cholesterol, LDL receptors are down‐regulated following the induced expression of the E3‐ligase MYLIP/IDOL 23, 24.

## Ubiquitin and receptor internalisation

**Figure 1:**
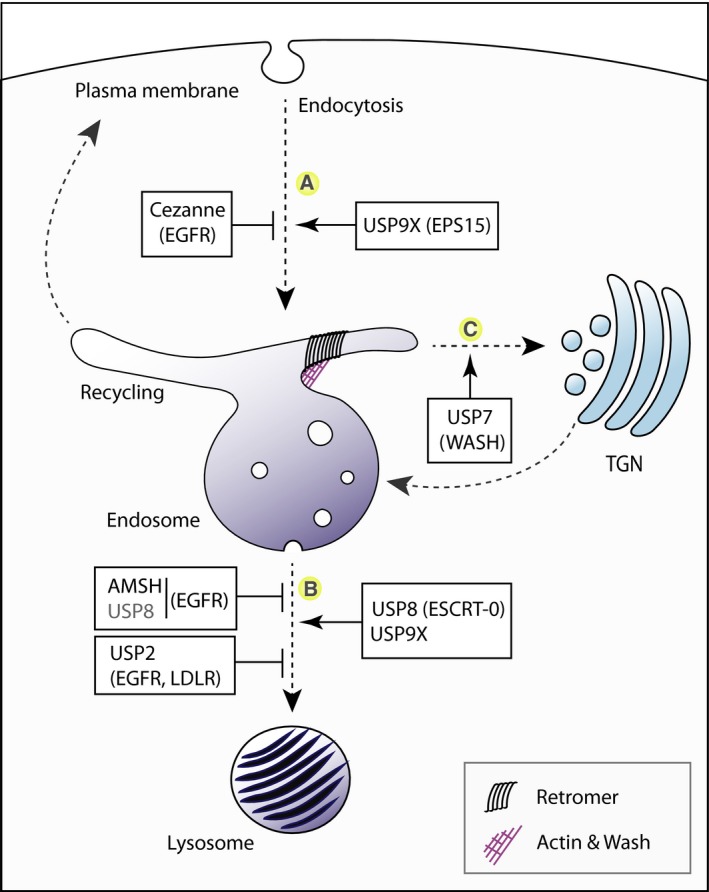
DUBs control trafficking through the endocytic pathway. Depicted are those DUBs that have been shown to affect (A) the internalisation process of a variety of transmembrane proteins (e.g. EGFR), (B) endo‐lysosomal sorting of cargo (e.g. EGFR) by the ESCRT‐machinery, leading to lysosomal degradation and (C) retromer and WASH‐dependent recycling of cargo proteins to the TGN (e.g. Mannose‐6‐Phosphate receptor). Proposed substrates are shown in brackets. Note that USP8 has been proposed to positively and negatively regulate endolysosomal trafficking, possibly reflecting its dual role in deubiquitylating both the ESCRT‐0 HRS/STAM complex and the EGFR itself. Dashed arrows show the path taken by cargo along the endocytic trafficking routes. Other DUBs implicated in endocytic trafficking of specific cargo, for example, USP10 and USP46 are not shown.

Ubiquitylation is one of several potential internalisation signals following receptor activation that allows coupling to adaptor proteins for both clathrin‐dependent and independent internalisation pathways 25. In the case of EGFR, a ubiquitin‐dependent nonclathrin pathway can be favoured under conditions of full receptor activation 26. A recent siRNA screen identified up to 15 DUBs influencing EGFR receptor turnover. Prominent positive regulators include USP6 and USP9X 27 (Fig. 1). Of these, USP6 showed the most pronounced plasma membrane localisation in a systematic survey of GFP‐DUB localisation 9. In a separate whole‐genome siRNA screen, USP6 was found to activate Wnt signalling by deubiquitylating Frizzled receptors and increasing their plasma membrane expression 28.

A number of accessory proteins associated with receptor internalisation have ubiquitin‐binding domains, but are themselves mono‐ubiquitylated in an EGF‐dependent manner (EPS15, EPS15R and Epsin) 29. USP9X depletion retards EGFR internalisation at low doses of EGF, when only the clathrin‐dependent pathway is operative, and little if any receptor is ubiquitylated. Savio and colleagues suggest that the relevant USP9X substrate is in fact the monoubiquitylated protein EPS15, but the mechanistic details of how this might influence internalisation are currently unclear 27. Studies in yeast suggest that deubiquitylation of the EPS15 homologue, Ede1 by Ubp2 and Ubp7, influences the rate of clathrin‐coated vesicle formation 30. In addition to its role in governing receptor internalisation, USP9X also plays a positive role in directing lysosomal trafficking from the sorting endosome 27. However, it does not directly influence the ubiquitylation status of the receptor itself, once again suggesting an indirect mechanism. Candidate substrates include the E3 ligase ITCH that associates with endosomes 31, or in fact an endosomal fraction of EPS15, which is ubiquitylated by the SPOPL/Cullin‐3 ubiquitin ligase 32. One further siRNA screen across the DUB family identified Cezanne as a negative regulator of EGFR down‐regulation 33.

## Regulation of endosomal sorting by DUBs

Endosomal sorting is accomplished through capture of ubiquitylated cargo at the endosome membrane by components of the Endosomal Sorting Complex Required for Transport (ESCRT) machinery 34, 35. This machinery promotes lumenal budding of cargo containing ILVs from the limiting membrane 36 and terminates growth factor receptor signalling by sequestration from the cytosol. It is classically divided into four subcomplexes, ESCRTs 0, I, II and III. Two DUBs, USP8 (also known as UBPY) and AMSH (STAMBP) are integrated within this complex protein network. The ESCRT‐0 complex, comprising HRS (HGS) and STAM, presents the first point of engagement of ubiquitylated receptors through multiple ubiquitin‐binding domains 34. Both DUBs engage with ESCRT‐0 by competing for a shared binding site on STAM. They also both contain N‐terminal MIT domains, which bind to overlapping spectra of multiple ESCRT‐III proteins 37, 38, 39. USP8 and AMSH belong to different subfamilies of DUBs; ubiquitin‐specific proteases and the metalloprotease JAMM family, respectively. AMSH has exquisite specificity for Lys63‐linked ubiquitin chains, while USP8 is nonselective 40, 41, 42, 43, 44. *In vitro*, both are activated through association with STAM, which binds to ubiquitin and decreases the Km 41, 45, 46.

As ubiquitin is a reversible post‐translational modification, a simple model posits that receptor fate at the endosome will be governed by competing ubiquitin‐conjugating and deubiquitylating activities 47. The Lys63 linkage‐specific DUB, AMSH, conforms to this model 41. Its depletion leads to enhanced degradation kinetics of acutely activated EGFR and its ligand 27, 33, 40, 48. Fusion of AMSH to the C‐terminus of EGFR blocks receptor degradation 18. AMSH is also recruited to GAP junction plaque sites and negatively regulates the turnover of the GAP junction protein Connexin 43 49. Note that the exquisite selectivity of AMSH for Lys63‐linkages will only allow for editing of ubiquitin chains, and that it cannot remove the proximal ubiquitin directly attached to receptors. Mutations in AMSH lead to Microcephaly Capillary Malformation Syndrome (MIC‐CAP) 50. These generally cluster in its MIT domain, but a catalytically inactivating mutation (T313I) has also been characterised 51.

USP2 has also been shown to localise to endosomes and negatively regulate EGFR down‐regulation 52. This enzyme will cleave all ubiquitin linkage types including the proximal ubiquitin. Loss of either protein provided a similar impact on EGFR degradation in the family‐wide DUB screen of Savio *et al*. 27. USP2 not only interacts with and stabilises the sterol‐regulated E3‐ligase IDOL but also rescues LDLR from lysosomal degradation, recalling the example of USP7 described in detail below 53. This principle of negative regulation of lysosomal sorting has been extended to many ubiquitylated receptors and channels and to other DUB family members. Examples include, the stabilisation of cystic fibrosis transmembrane conductance regulator (CFTR) by USP10 54 and glutamate receptors in both nematode and mammalian model systems by USP46 55, 56.

The effects of USP8 depletion are profound and widespread, leading to a major disruption of endosomal organisation. In distinction to AMSH, USP8 depletion predominantly impairs RTK degradation, but in addition blocks the retromer‐dependent retrieval of the cation‐independent mannose 6‐phosphate receptor (ciM6PR) to the Golgi apparatus and the plasma membrane recycling of the VEGFR2, Frizzled (Fz) and Smoothened (Smo) 27, 45, 57, 58, 59, 60, 61. One major consequence of USP8 depletion is the loss of both ESCRT‐0 components, HRS and STAM, which are turned over in a proteasome‐dependent manner 45, 62, 63. Despite binding to the same site on STAM and being expressed at similar levels 5, AMSH cannot compensate for USP8 loss owing to its restricted ubiquitin chain specificity 64. In addition, USP8 governs the global flux of cellular ubiquitin; its depletion drains the nucleus of ubiquitin by accumulation on endosomes 45, 60. Note that the pleiotropic effects of USP8 make the observed phenotypes very sensitive to the efficiency of depletion in siRNA experiments, as explicitly addressed by Mizuno *et al*. 65. Certain scenarios, for example, suboptimal depletion or particular cell types, reveal negative regulation of lysosomal trafficking by USP8 66, 67 (Fig. 1). Overexpression of USP8 also leads to enhanced recycling of the epithelial Na^+^ channel 68. Activating mutations in USP8 have been linked to Cushing's Disease, which is caused by corticotroph adenomas of the pituitary 69, 70. One suggestion is that this may reflect increased levels of EGFR through enhanced recycling to the plasma membrane.

The effect of USP8 on the retromer pathway may be linked to its control of HRS stability. HRS has been suggested to interact with the core retromer components SNX1 and VPS35 and to influence this pathway 71. The retromer‐dependent pathway requires the participation of F‐actin which is recruited by the WASH complex. Activity of WASH is positively regulated by ubiquitylation, mediated by an E3 ligase complex of TRIM27 and MAGE‐L2 72. Hao *et al*. have reported that USP7 incorporates into this complex through interactions with both components and localises to endosomes (Fig. 1) in addition to the nucleus where it has previously established roles 73. USP7 has opposing activities in the context of the retromer pathway; it stabilises the WASH E3‐ligase TRIM27 by opposing its auto‐ubiquitylation, but it also deubiquitylates WASH itself. It is proposed that USP7 is used to buffer WASH ubiquitylation to maintain optimal endosomal activity and F‐actin levels. Interestingly heterozygous deletion or mutation of USP7 leads to mental impairments that resemble Schaaf‐Yang syndrome which is associated with MAGE‐L2 mutations 73.

**Figure 2:**
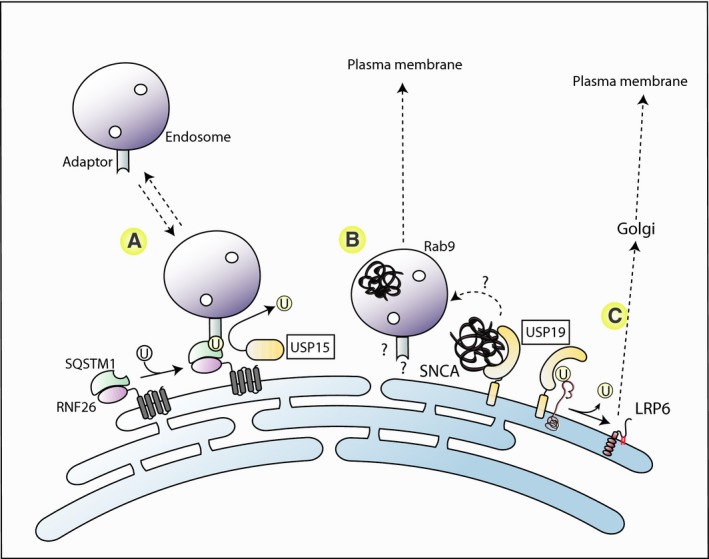
DUB activities associated with endoplasmic reticulum (ER)‐associated vesicles. (A) The integral ER protein RNF26 is proposed to ubiquitylate SQSTM1/p62, which in turn functions to tether a variety of vesicles including endosomes via endosomal ubiquitin‐binding adaptors, in the perinuclear area. USP15, which is a cytoplasmic protein is thought to release the vesicles by deubiquitylating SQSTM1. (B) The ER transmembrane protein USP19 has been suggested to act as a chaperone to promote the transfer of misfolded alpha‐synuclein (SNCA) into the lumen of RAB9‐positive late endosomal vesicles, which subsequently exocytose their content by fusion with the plasma membrane via a noncanonical MAPS pathway. (C) USP19 engages in a number of quality control pathways, including ERAD (not shown) and the ubiquitin‐dependent ER‐exit of the palmitoylated and glycosylated LRP6 transmembrane protein via vesicular carriers.

There is an emerging appreciation that distinct organelles can influence each other at sites of direct contact. For example, the ER‐localised phosphatase, PTP1B, can dephosphorylate the EGF receptor on endosomes 74. The closely related DUBs, USP20 and USP33, deubiquitylate activated β‐adrenergic receptors as well as the adaptor/signalling protein β‐arrestin (in the case of USP33), to favour recycling from endosomes, despite largely being associated with the secretory pathway 75, 76, 77. In a further intriguing model, the ER restricted E3‐ligase RNF26 is proposed to bind to and ubiquitylate SQSTM1/p62. This then links to ubiquitin‐binding adaptors (e.g. EPS15, TOLLIP) on endosomal vesicles to tether them on the ER and confine them to perinuclear regions (Fig. 2) 78. It is unclear how this relates to an existing model suggesting that SQSTM1 links cargo to the (‐) end directed microtubule motor protein, dynein, that drives inward movement of endosomes 79. USP15, which has also been identified as a binding partner of RNF26, reverses this capture by deubiquitylating SQSTM1, thereby increasing endosome mobility and global distribution 78.

## Ubiquitylation, a key mediator of selective autophagy

Early studies of autophagy emphasised its role as an adaptive response to amino acid deprivation, through the indiscriminate breakdown of cytosolic components. It is now clear that many modes of selective autophagy exist and that in most, but not all cases, accumulation of ubiquitin provides the means to recruit adaptor proteins, which bridge to LC3/GABARAP‐coated phagophore membranes 80. It seems likely that multiple widely distributed ubiquitin‐binding sites on the target body will favour coupling to autophagosomes rather than proteasomes using the co‐operativity between multiple weak binding interactions of adaptors for both ubiquitin and LC3. In most cases it would seem that a relatively delocalised coating with ubiquitin is more critical than ubiquitylation of specific protein substrates. DUBs may act to limit this distress signal or alternatively act to stabilise core components of the autophagy pathway. For example, USP9X and USP10 have been proposed to regulate the stability of VPS34 complex components 81, 82. Autophagosomes then fuse with lysosomes to generate autolysosomes. Autophagic flux is therefore reliant on appropriate organisation of the endo‐lysosomal system.

Several studies have highlighted a role for USP8 in various aspects of autophagy, mirroring its complex contribution to endosomal functions (see above). A screen in the Drosophila larval fat body for DUBs influencing autophagy identified USP12 and USP8 as regulators of autophagic flux. In larval fat body cells depleted of USP8, autophagic degradation is blocked, presumably owing to the associated lysosomal defects. In contrast, when the authors turned to HeLa cells they found that depletion of USP8 increased the number of intracellular punctae decorated with the autophagic marker GFP‐LC3, with a proportionate increase in the autophagic flux 83. The accumulation of α‐synuclein (SNCA) in neuronal inclusions known as Lewy bodies is a hallmark of Parkinson's Disease (PD). Recent data suggest that USP8 may negatively regulate degradation of α‐synuclein by removal of Lys63 ubiquitin chains. At this point it is not clear if this represents a deflection from the autophagic pathway or from capture of soluble protein into MVBs. Most strikingly a rough eye phenotype in Drosophila, induced by α‐synuclein expression can be reversed by USP8 depletion 84.

## Mitophagy

**Figure 3:**
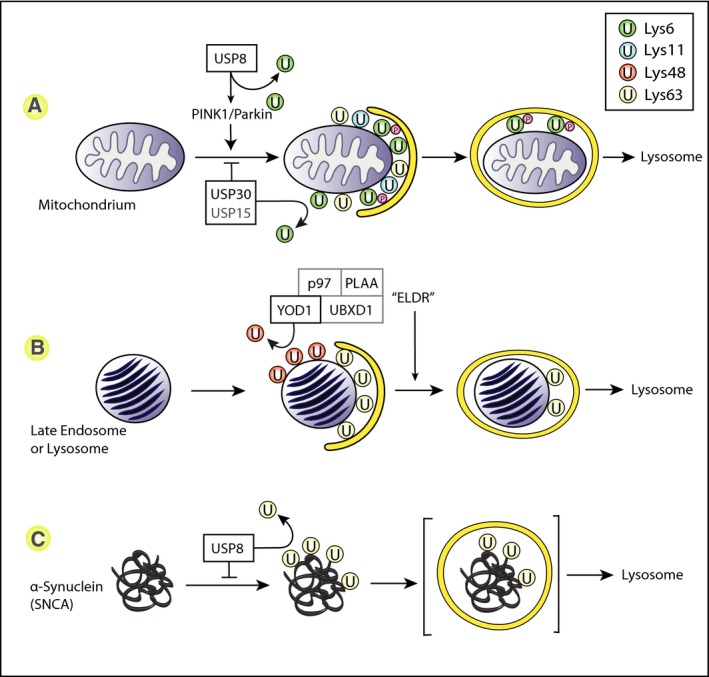
Selective autophagy is regulated by mutiple DUBs. (A) Mitophagy is triggered by the activation of PINK1 and Parkin leading to the accumulaton of Lys6, Lys11‐ and Lys63‐linked Ub chains, containing pUb, on the outer mitochondrial membrane. These promote the association of ubiquitin‐binding adaptor proteins that recruit LC3‐decorated autophagic membranes. USP30 and USP15 have been proposed to counteract this process by removing ubiquitin from Parkin substrates. USP8 has been proposed to remove inhibitory Lys6‐linked ubiquitin chains from Parkin. (B) The endo‐lysosomal damage response ensures the clearance of damaged lysosomes via autophagy. Lys63‐linked chains are proposed to act as an enabling ubiquitin modification in this process and promote the recruitment of the autophagic machinery. In contrast, Lys48‐linked chains associated with some of the damaged lysosomes need to be removed by the DUB YOD1, a component of the Endo‐Lysosomal Damage Response complex (ELDR). (C) Misfolded alpha‐synuclein aggregates have been shown to accumulate Lys63‐linked ubiquitin chains which promote its degradation within lysosomes. USP8 is proposed to remove these ubiquitin chains and interfere with the clearance of alpha‐synuclein aggregates.

Defective mitochondria can depolarise and accumulate the damage sensor PTEN‐induced putative kinase 1 (PINK1) on their surface 85. This enables recruitment of the E3‐ligase Parkin to the surface of mitochondria. Loss of function mutations in PINK1 and Parkin lead to PD. Full activation of Parkin requires both its own phosphorylation by PINK1 and phosphorylation of ubiquitin on Ser65 (pUb). PINK1‐generated pUb acts as a Parkin receptor on mitochondria and elicits a conformational change which is stabilised by PINK1‐dependent phosphorylation of an internal Ubl domain 86, 87, 88 (Fig. 3). This leads to clearance of mitochondria via the autophagy pathway utilising specific adaptor proteins, optineurin and NDP52 85, 89, 90, 91.

USP30 is the major mitochondrial DUB, facing the cytosol and anchored to the outer membrane through a trans‐membrane sequence 92. It is optimally placed to reverse the Parkin‐dependent ubiquitylation of multiple mitochondrial surface proteins and thereby limit mitophagy 93, 94. Mitochondrial damage stimulates Parkin to generate Lys6, Lys11 and Lys63‐linked polyubiquitin chains, while USP30 preferentially disassembles the Lys6 and Lys11‐linked multimers. However, USP30 is less active towards pUB chains 95, 96, 97, 98. One model consistent with this observation envisages that by suppressing basal ubiquitylation at mitochondria, USP30 may effectively limit PINK1 ubiquitin‐substrate availability and the generation of pUb ‘Parkin‐receptor sites’, thus primarily influencing the initiation phase of mitophagy 86.

USP30 overexpression impairs Parkin recruitment to depolarised mitochondria and blocks mitophagy 85, 99. Conversely, its depletion enhances mitophagic flux in cultured neurons and epithelial cells 85, 99. In PINK1/Parkin defective, or paraquat‐treated fly models of PD, depletion of USP30 can restore motor function and dopamine levels 85. Reversal of Parkin ubiquitylation at mitochondria primarily reflects the specific localisation of USP30, since targeting other USP family members to mitochondria can fulfil the same function and directing USP30 to peroxisomes protects them from pexophagy 97.

Similar attributes have been suggested for USP15, which is more widely distributed throughout the cytoplasm and associated with diverse cellular functions. Overexpression of USP15 reduced ubiquitin accumulation on mitochondria, while depletion rescued mitophagy defects in PD patient‐derived fibroblasts. Concomitant depletion of a proposed Drosophila USP15 orthologue mitigated the effects of Parkin knock‐down by siRNA 100. It is presently unclear, how direct the effect of USP15 may be. Cornelissen *et al*. first focused on USP15 after identifying it as coimmunoprecipitating with His6‐FLAG‐tagged Parkin, but other extensive Parkin interactome studies have not found a similar interaction 100, 101, 102. A short form of USP35 (s‐USP35) has also been localised to mitochondria and is suggested to regulate mitophagy 99. However, note that this form of the enzyme lacks a catalytic domain. USP8 is proposed to have a nonendosomal function in controlling Parkin autoubiquitylation with predominantly Lys6‐linked chains. By virtue of this activity, Parkin recruitment to acutely depolarised mitochondria is accelerated and mitophagy proceeds more efficiently 103.

## Endo‐lysosomal damage response (ELDR)

Endosomal compartments may get damaged by endogenous factors including ROS and neurotoxic aggregates, in the course of pathogen entry and experimentally, through the application of various transfection reagents 104. This leads to ubiquitylation and recruitment of the autophagic machinery via specific adaptor molecules such as Atg16L1 105. Papadopoulos *et al*. described a role for the AAA‐ATPase p97 in the efficient clearance of damaged endosomal compartments by selective autophagy, following the application of a lysosomotropic agent. Three p97‐interacting proteins, UBXD1, PLAA and the DUB YOD1/OTUD2, were also required for the clearance of damaged lysosomes. A positive requirement for a DUB in autophagic clearance is unexpected. They suggest that a subset of Lys63 ubiquitin chain‐decorated endosomes also accumulate Lys48‐linked chains which need to be removed by YOD1 to allow envelopment by LC3 containing phagophores 106. *In vitro*, YOD1 discriminates between these chain types, although Lys 27, 29 and 33 chains are preferred substrates 44.

## Ubiquitin and the secretory pathway

The major function of ubiquitin, heretofore associated with the secretory pathway is mediation of the ER‐associated degradation (ERAD) pathway, which has been extensively reviewed elsewhere 107. A role of nonspecified DUB activity has been proposed in sharpening the discrimination of triaged ERAD substrates, much as previously described for both endosomal‐ and proteasome‐associated DUBs 108. In preceding studies the ER‐localised USP19 was shown to rescue the ERAD substrates cystic fibrosis transmembrane conductance regulator (CFTR)ΔF508 and T‐cell receptor‐α (TCRα) from proteasomal degradation 109. Several DUBs associate with the retro‐translocation‐driving ATPase p97 (e.g. YOD1, ATXN3, USP13), some of which can positively regulate the ERAD pathway 7, 110, 111, 112.

Some DUBs show clear association with organelles of the secretory pathway, yet distinct roles in membrane trafficking have not been reported. One exception is the isoform of USP19, which contains a *trans*‐membrane anchor that exclusively targets it to the ER 9, 109. Recent work has associated it with an unconventional secretion pathway that becomes important under conditions of proteasome depletion or disfunction (Misfolding Associated Protein Secretion, MAPS). The catalytic domain of USP19 is proposed to capture misfolded protein (including α‐synuclein) at the surface of the ER and facilitate their transfer into vesicular structures that contain late endosomal markers (e.g. rab9), but are distinct from exosome carriers. This property of USP19 requires both ER localisation and catalytic activity. The resultant carrier vesicles then secrete proteins after fusion with the plasma membrane 113. A further “chaperone like” activity of USP19 has also recently been proposed. Folding of the Wnt signalling coreceptor LRP6 is promoted by ubiquitylation of a specific cytosol facing lysine which promotes its retention in the ER. Exit from the ER of folded protein is then enabled by USP19‐mediated deubiquitylation 114 (Fig. 2).

Other functions of ubiquitin associated with vesicle trafficking and compartmental identity are beginning to emerge. Properly folded secretory proteins are packaged into COPII vesicles for transport from the ER to the *cis*‐Golgi. Early studies in yeast suggested that a complex of Bre5 and the DUB Ubp3 regulate this process, possibly by sustaining the levels of the COPII coat component Sec23p 115. Some secreted proteins such as collagen and fibronectin are too large to be included in a standard size vesicle, presenting a long‐standing conundrum to the field 116. Jin *et al*. have shown that mono‐ubiquitylation of the COPII component Sec31p permits expansion of vesicle size and increased secretion of collagen. The relevant E3 ligase has been identified as a cullin‐RING ubiquitin ligase (CRL) complex incorporating the BTB protein KLHL12 as a recognition module in conjuction with two penta‐EF‐hand calcium‐binding proteins PEF1 and ALG2 which couple a transient rise in Ca^2+^ to a more sustained ubiquitin signal on Sec31p 117, 118. Secretion of another large protein fibronectin is controlled by the cytosolic CRL incorporating von‐Hippel Lindau (VHL) adaptor subunit 119.

The related DUBs, USP20 and USP33, are also present on the ER. They have both been linked to the maintenance of activity of the ER associated type 2 iodothyronine deiodinase that activates thyroxine 120. Both also interact with VHL but only USP20 appears to influence its stability 121. USP33 also resides on COPII vesicles and on the Golgi Apparatus. Its distribution can be shifted according to which splice variant is expressed, such that variant 3 selectively accumulates on the Golgi apparatus 77.

During mitosis, the Golgi complex fragments in order to achieve an equal distribution of organelle between progeny cells. Reassembly requires SNARE proteins, in common with the majority of intracellular membrane fusion events 10. A fraction of the Golgi t‐SNARE, syntaxin 5 is monoubiquitylated by the E3‐ligase HACE1 in early mitosis leading to a block in the interaction with its cognate v‐SNARE Bet1 122, 123. It concomitantly increases interaction with the p97/p47 complex, which then promotes postmitotic Golgi assembly following ubiquitin removal by its interacting DUB VCIP135 123, 124, 125, 126. Temporal control is strengthened through phosphorylation‐dependent inactivation of VCIP135 early in mitosis and reversal by phosphatase activity late in mitosis 126, 127.

Anterograde trafficking from the *Trans*‐Golgi Network routes cargo to the plasma membrane or to the lysosome. Studies with mutant ubiquitin molecules suggest distinct roles for Lys33‐ and Lys63‐linked chains in post‐Golgi trafficking 128. The CUL3‐KLHL20 E3‐ligase promotes Lys33‐linked ubiquitin chain formation on Coronin 7 which is then recruited by a Golgi‐localised fraction of EPS15. This is proposed to promote F‐actin assembly which in turn favours the budding of transport carriers destined for the plasma membrane (e.g. VSV G protein) or the endo‐lysosomal pathway (e.g. ciM6PR) 128.

## Concluding remarks

Deubiquitylating enzymes are most simply conceived as negative regulators of ubiquitin‐dependent processes. This holds true for their control of several ubiquitin‐mediated protein‐sorting events, where they may directly deubiquitylate cargo proteins. Similarly, we also expect more DUBs to be discovered that dampen signals for selective autophagy events, in addition to USP30. However, the preceding text also highlights positive roles for DUBs in more complex pathways, which might require cycles of ubiquitylation and deubiquitylation. Several generic reasons for this may be envisaged: (a) ubiquitin chain editing to switch or harmonise a linkage type, (b) release from a ubiquitin receptor, (c) control of stability of cognate E3‐ligases by opposing their autoubiquitylation, (d) control of stability of other accessory factors.

The current survey illustrates that a significant fraction of the ~ 100 DUB components of the ubiquitin system impact upon the cellular membrane trafficking system. However, several specific membrane trafficking events have been shown to be ubiquitin‐dependent, without relevant DUB activities so far being characterised. Some DUBs show distinct subcellular distributions, without a clear understanding of their localised function. DUBs help shape the cellular architecture, but then use this foundation to influence further systems, such as cell signalling. For example, since USP30 can influence mitochondrial dynamics, we might further expect it to influence ubiquitin‐dependent signalling systems associated with mitochondria. To progress our understanding of the ubiquitin system we will need to have an idea of its demographics (the abundance of each functional component 5) as well as its geography (distribution and landscape).

## Author contributions

MJC and SU wrote the review and prepared the figures.
